# Effect of* Bletilla striata* on the Prevention of Postoperative Peritoneal Adhesions in Abrasion-Induced Rat Model

**DOI:** 10.1155/2019/9148754

**Published:** 2019-06-10

**Authors:** BoTong Liu, QiuHua Zhang, Xiao Wu, YanJun Fu, Hui Wang, YunXia Guan, ShiCheng Yang, YiLan Liu, WenCong Cao, Jian Wang

**Affiliations:** ^1^Department of Basic Pharmacology, Liaoning University of Traditional Chinese Medicine, 110032 Shenyang, China; ^2^Department of Animal Experimental Center, HE's University, 110163 Shenyang, China; ^3^Department of Laboratory Animal Center, Liaoning University of Traditional Chinese Medicine, 110032 Shenyang, China

## Abstract

Postoperative peritoneal adhesions (PPAs) constitute a common complication of abdominal surgery with a high incidence.* Bletilla striata *(BS) is an important hemostatic drug used in China for nearly 2000 years. The purpose of this study was to investigate the effect of* Bletilla striata* on postoperative intestinal adhesion in rats. PPA was induced by cecal wall abrasion, and* Bletilla striata* was injected to observe its effect on adhesion in rats. The adhesion and inflammation score were assessed through visual observation and histopathologic evaluation. The levels of interleukin-1 (IL-1*β*), tumor necrosis factor (TNF-*α*), and interleukin-17F (IL-17F) in abdominal cavity and interleukin-6 (IL-6) in plasma were measured by enzyme-linked immunosorbent assay (ELISA) at 6 hours, 12 hours, 24 hours, and 1 week after operation. The tissue level of transforming growth factor beta-1 (TGF-*β*1) was also determined by ELISA on the seventh day after surgery. The expressions of collagen and TNF-*α* were, respectively, detected by Masson trichrome staining and immunohistochemical staining. The expression of TGF-*β*1 and alpha smooth muscle actin (*α*-SMA) was detected by Western blot. The result showed that* Bletilla striata* has obvious preventive effect on PPAs and celiac inflammation of PPAs.* Bletilla striata* could significantly reduce the level of IL-17F abdominal cavity and IL-6 in plasma. Masson trichrome staining and immunohistochemical staining results showed that* Bletilla striata* also decreased the expression of TNF-*α* and collagen. Western blot results showed that* Bletilla striata* decreased the expression of *α*-SMA and TGF-*β*1. Our results suggest that* Bletilla striata *decreased the development of abdominal adhesion in abrasion-induced model of rats and reduced the expression of the important substance which increased in PPAs.* Bletilla striata* can be further studied as a new and cheaper antiadhesive substance.

## 1. Introduction

Postoperative peritoneal adhesions (PPAs) constitute a common complication of abdominal surgery; the incidence rate is up to 90%-95% [1]. PPAs may cause significant morbidity, including adhesive small bowel obstruction, female infertility, chronic abdominal pain, and increased difficulty with subsequent surgery [2, 3]. The formation of PPAs involves a complex interaction of cytokines, growth factors, cell adhesion molecules, neuropeptides, and numerous other factors secreted by cells in or near the area of trauma [4, 5]. Preventive measures include keeping the lesions separated, increasing bowel motility, and decreasing local inflammation [6]. Currently, there is no widely accepted method of treating abdominal adhesions, so we still need to find a more effective treatment method to prevent abdominal adhesion after operation.

*Bletilla striata* (Thunb.) Reichb.f. (Orchidaceae) is an important astringent hemostatic medicinal plant used for almost 2000 years in China and is widely distributed in eastern and southern Asia. Many studies have demonstrated that it exhibits wound healing activity, antiulcer activity, hemostatic activity, and anti-inflammatory activity [7]. Recently, a research confirmed its protective effect on renal fibrosis, which is probably mediated by downregulated alpha smooth muscle actin (*α*-SMA) in vitro. [8]; therefore, we speculate that* Bletilla striata* can prevent abdominal adhesion after operation.

Although the mechanism of abdominal adhesion after operation is not very clear, it represents exaggerated and dysregulated peritoneal “repair” mechanisms in response to trauma, leading to a series of local responses involving acute inflammation, extracellular matrix (ECM) deposition, fibrinolysis, and neoangiogenesis [9, 10]. Therefore, the local inflammation and transforming growth factor (TGF)-*β* family are the important factors of abdominal adhesion after operation. Hence, the main purpose of this study was to investigate the effect of* Bletilla striata* on abdominal adhesions, inflammatory factors and related fibrogenic factors such as TGF-*β*1 in the cecum tissue to verify our hypothesis that the inhibitory effect of* Bletilla striata *on PPAs is closely related to the inhibition of TGF-*β*1 and* Bletilla striata* can be further studied to develop into a therapeutic drug for the prevention of abdominal adhesions.

## 2. Materials and Methods

### 2.1. Chemicals and Biochemicals

The ELISA Kits of IL-1*β* (EK0393), IL-6 (EK0412), TNF-*α* (EK0526), and IL-17F (EK1136) were purchased from the Boster Biological Co., Ltd. The ELISA Kit of TGF-*β*1 (m10021856) was purchased from Mlbio Biological Co., Ltd. HRP AffiniPure Goat Anti-Rabbit IgG (E030120) and GAPDH (E021060-03) were from EarthOx Life Sciences. SDS-PAGE loading buffer (5X) (P0015), Western and IP lysis buffer (P0013), ECL luminous solution (P0018), Primary and Secondary Antibody Dilution Buffer (P0023A, P0023D), BCA Kit (P0012S), and SDS-PAGE Gel Kit (P0012A) were purchased from Beyotime Biological Co., Ltd. *α*-SMA monoclonal antibody (#19245) and TNF-*α* polyclonal antibody (WL01896) were, respectively, from Cell Signaling and Wanleibio Limited Companies. Protease Inhibitor Cocktail (A8260) and Histostain-SP Kits (SPN-9001) came from Solarbio Science and Technology Co., Ltd. and Zsbio Commerce Store.

### 2.2. Extraction of Plant Material

*Bletilla striata *was purchased from Department of Pharmacy, Affiliated Hospital of Liaoning University of Traditional Chinese Medicine, and was identified as the original plant of* Bletilla striata* (Thunb.) Rchb.f. by the professor Wang Weining, Liaoning Institute of Pharmacology. An amount of 150g* Bletilla striata* was collected for drying, crushing, and cold soaking before aqueous extraction; then 1000 ml distilled water was added to boil for 45 min. After collecting the filtrate, the residue was boiled twice for 30 min with 500ml distilled water. Subsequently, three times of filtration solution evaporated and condensed into 50ml decoction, then the decoction was added to ethanol, refrigerated, filtered, and recovered. The decoction was added to injection water and 2% activated carbon to 1000ml and boiled for 30 min, filtered and packed in a bottle, and sterilized for further usage.

### 2.3. Animals and Diets

80 male Sprague-Dawley rats (Liaoning Changsheng Biotechnology co., Ltd.) weighing 169~206g were acclimated for one week before the experiments. They were housed in controlled room (temperature: 20-25°C, relative humidity: 40%-70%, air circulating frequency: 20 times/hour, artificial light: 15-20Lux from 8:00 AM to 8:00 PM, noise: <60 db). Rats were given standard rat chow and free access to water. This experiment was approved by the ethical test of animal experiment of Liaoning University of Traditional Chinese Medicine (No. 21000092017048).

### 2.4. Study Design

The adhesion model in this study is based on the adhesion model described by Hemadeh et al. [11]; the method can cause 100% abdominal adhesion in rats 7 days after abdominal operation. Rats were only provided with water before the operation and weighed and anesthetized by intramuscular injection of chloralic hydras, breathing spontaneously throughout the procedures. The abdominal skin of rats was shaved and cleaned with povidone-iodine solution. The operation was performed under aseptic conditions. In each rat, a 3 cm vertical midline incision was made, and then the abdomen was opened. Except for the sham operation group, the caecum of rat in the three groups was exposed to the ventral and dorsal surfaces and gently rubbed with 2 dry gauze pieces until the gloss and bleeding point appeared, and the caecum was recovered to the anatomic position. Sham operation group was subjected to midline incision of the abdomen without any scratch. After abdominal closure, animals were randomly divided into 4 groups with 32 animals in each group. The rats in the sham operation group was not treated; the other rats of the three experimental groups were intraperitoneally injected with 8 ml of phosphate-buffered saline (Control group), 15%* Bletilla striata *extraction solution (BS group), and 0.2% hyaluronic acid solution (HA group), respectively. The concentration of HA was similar to that of Reijnen et al. [12] as a positive control group. The dose of* Bletilla striata* is lower than the minimum dose of human-rat conversion as stipulated in the Chinese Pharmacopoeia 2015, which means that the dose of* Bletilla striata* used for rats is the safe dose.

All animals were given water only on the first day and given normal diet and water on the second day after operation. A week later, all rats were euthanized and the abdomens of the rats were opened through a U-shaped incision, thoroughly explored.

### 2.5. Adhesion Grading and Evaluation

Methods described by Nair et al. [13] were used to determine the degree of adhesion on the 7 days after operation. This scoring system is based on the following criteria: 0 represents the presence of no adhesive bands; 1 represents a single adhesive zone between the viscera or between the viscera and the abdominal wall; 2 represents two bands formed between the viscera or between the viscera and the abdominal wall; 3 represents more than two bands formed between the viscera or between the viscera and the abdominal wall, or the whole intestine formed a mass without attachment to the abdominal wall; 4 indicates that the viscera are directly attached to the abdominal wall without being affected by the number of bands.

### 2.6. Sample Collection

Blood was collected through abdominal aorta by vacuum tube (with heparin). Peritoneal fluid was perfused with 10 ml PBS. Samples were transferred to centrifuge at 4°C (120×g) for 3 min and stored at −80°C until analysis. The adhesive caecum was carefully removed, and the tissue was cut longitudinally to remove contents and washed with aseptic PBS. Half of the tissues in each group were fixed in 10% formalin in PBS for histopathologic evaluation, immunohistochemistry, and Masson trichrome staining. The other half of tissues in each group were stored at -80°C for ELISA and Western blot analysis.

### 2.7. Histopathologic Evaluation

The cecal was fixed in 10% formalin in PBS for at least 1 hour. After routine processing, serial sections were stained with hematoxylin and eosin. The other sections were stained with Masson trichrome stain. The degree of inflammation was evaluated in a double-blind study by two assessors using a scoring system described by GD et al. [14]: grade 1, macrophages, occasionally scattered around lymphocytes and plasma cells, indicate a mild inflammatory reaction; grade 2, macrophages increased and admixed with lymphocytes, plasma cells, eosinophils, and neutrophils, mean a moderate reaction; grade 3, the presence of microabscesses means a severe inflammatory response.

### 2.8. Enzyme-Linked Immunosorbent Assays (ELISA)

The concentrations of IL-*β*1, IL-6, TNF-*α*, and IL-17F in peritoneal fluid and IL-6 in plasma were determined by ELISA kit. The concentration of TGF-*β*1 in cecum tissue was detected according to the manufactures' instructions.

### 2.9. Immunohistochemistry

Immunohistochemical staining was performed by using a Histostain-SP Kits following the manufacturer's instructions. The sections were deparaffinized and rehydrated, then incubated with 30 g/L 3% hydrogen peroxide solution at room temperature for 10 minutes, and washed 5 times in distilled water. After antigen repair, the sections were incubated in blocking buffer. These sections were incubated overnight at 4°C with antibody TNF-*α*. After the section was washed repeatedly, biotinylated secondary antibody working solution and horseradish peroxidase-conjugated streptavidin working solution were added, respectively. The slices were washed in PBS (pH 7.2) for 5 minutes. Diaminobenzidine tetrahydrochloride (DAB) was used for visualization, and hematoxylin was used as the counterstain; the sections were dehydrated, mounted, and sealed. An image signal acquisition and analysis system (Leica) was used for image acquisition. Positive cells were counted as a brown deposit in cells, made in randomly selected high power fields (400×; 2.37 mm^2^) [15].

### 2.10. Western Blotting

Western blotting was prepared according to the procedure used by Guo et al. [16]. The cecum or peritoneum of rats was placed in a lysis buffer containing 10 u L phosphatase inhibitor, 1 u L protease inhibitor, and 5 u L 100 mM PMSF. After boiling for 5 min, 75 micrograms of protein was dissolved in the loading buffer and denatured. Each sample was subjected to electrophoresis on 10% SDS polyacrylamide gel. Then the protein was transferred to the PVDF membrane. After blocking the membrane for 1 h at room temperature with blocking buffer, the membranes were probed with primary antibodies including TGF-*β*1 (1/1000 dilution), *α*-SMA (1/1000 dilution), and GAPDH (1/5000 dilution) as appropriate with constant shaking overnight at 4°C. After washing with 0.1% Tween-20 in TBS for three times, the secondary antibody was incubated in the buffer at room temperature for 1 h. The bound antibodies were detected with enhanced chemiluminescence system (Millipore) as recommended by the manufacturer.

### 2.11. Bioinformatics

We used TCMID, the world's largest database of traditional Chinese medicine ingredients, to search for the active ingredients of* Bletilla striata* [17]. Then we used BATMAN-TCM to analyze the biopathway enrichment of the active ingredients of* Bletilla striata* in the Kyoto Encyclopedia of Genes and Genomes (KEGG) [18]. The significantly enriched paths of the active ingredients of* Bletilla striata* were chosen to draw the bar chart (P < 0.05).

### 2.12. Statistical Analysis

Quantitative data were expressed as the mean ± SEM. The Kruskal-Wallis test was used to observe the degree of the adhesion and inflammatory degrees. One-way ANOVA analysis was used to evaluate the differences among the four groups. Fisher's least significant difference (LSD) test was used to determine the differences between each group. The significance level was set at P < 0.05. Statistical analysis was performed using SPSS 19.0 software.

## 3. Results

### 3.1. *Bletilla striata* Reduced Abdominal Adhesion Scores in Rats

**Figure 1:**
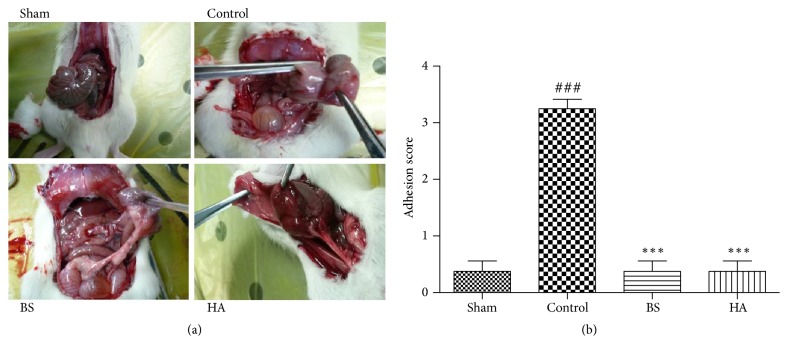
*Bletilla striata* reduced postoperative intestinal adhesion in rats. (a) The typical image of abdominal adhesion in each group rats. Sham operation group: no adhesions; control group: the whole cecum forms a mass without adhering to the abdominal wall; BS and HA group: a single adhesion band formed between the viscera or between a viscus and the abdominal wall. (b) Adhesion scores in each group, ###P<0.001 compared with sham laparotomy group. *∗∗∗*P<0.001 compared with control group.

128 male Sprague-Dawley rats completed the whole experiment, and no death occurred among the four groups of rats.  Figure 1(a) is a typical image of abdominal adhesion in rats of each group. Based on the Nair's scoring system, the average adhesion score per group is shown in Figure 1(b). The adhesion score of the model group was significantly higher than that of the sham operation group (P < 0.001), which proved that the model was made successfully. The adhesion score of* Bletilla striata* group and HA group was significantly lower than that of model group, indicating that* Bletilla striata* and HA could inhibit PPAs.

### 3.2. *Bletilla striata* Alleviates the Inflammatory Reaction of PPAs in Rats

**Figure 2:**
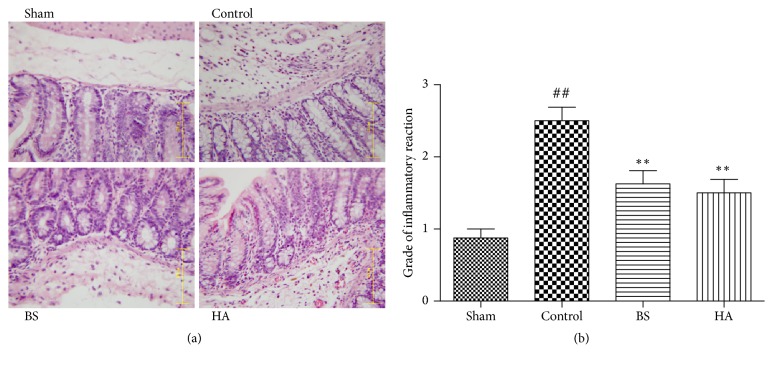
*Bletilla striata* alleviates the inflammatory reaction of postoperative intestinal adhesion in rats. (a) The typical image of inflammatory response among sham laparotomy, control, BS, and HA group by H.E. (400×). (b) The grade of inflammatory reaction in each group (n =8). *∗∗*P<0.01 compared with the control group. ##P<0.01 compared with the sham group.

In our study, we observed that the inflammatory reaction of PPAs mainly occurred in the mucosa, submucous membrane, and muscle layer by the abdominal adhesion, H.E staining, and immunohistochemical staining. The control group showed increased admixed lymphocytes, plasma cells, eosinophils, and neutrophils (inflammation grade 3) (Figure 2(a)). Compared with the control group, the hyperemia and inflammatory reaction of rats treated with* Bletilla striata* and HA were obviously alleviated (Figure 2(b)); the scores of inflammatory response in BS group and HA group were significantly lower than those in control group (Figure 2(b)); the results showed that* Bletilla striata* alleviates the inflammatory reaction of PPAs in rats.

### 3.3. *Bletilla striata* Reduces the TNF-*α* Expression in Caecum on the 7th Day after Operation

**Figure 3:**
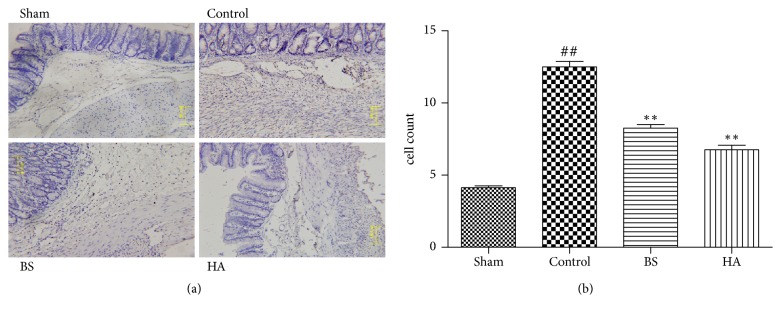
*Bletilla striata* reduces the TNF-*α* expression in caecum on the 7th day after operation. (a) The typical image of TNF-*α* expression among sham laparotomy, control, BS, and HA group by immunohistochemical staining. (b) Analysis of TNF-*α* cell count by immunohistochemical staining, *∗∗*P<0.01 compared with the control group. ##P<0.01 compared with the sham group.

TNF-*α* immunohistochemical staining in abdominal adhesion tissue showed that the cell numbers of TNF-*α* expressing in control group were significantly higher than that in the sham operation group. The expression of TNF-*α* in caecum of rats treated with* Bletilla striata* and HA was significantly lower than that of control group (P < 0.01) (Figure 3); the results showed that* Bletilla striata *inhibited the TNF-*α* expression in caecum on the 7th day after operation.

### 3.4. *Bletilla striata* Inhibited the Levels of IL-6 in Plasma and IL-17F in Peritoneal Fluid after PPAs in Rats

**Figure 4:**
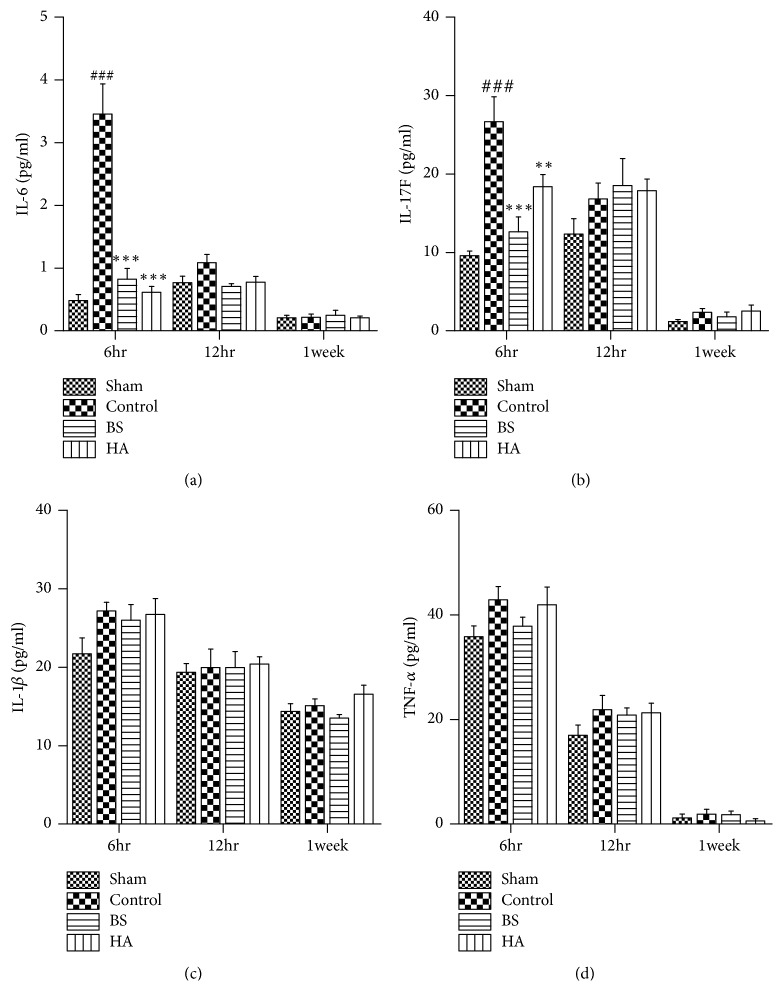
*Bletilla striata* inhibited the levels of IL-6 in the plasma and IL-17F in the peritoneal fluid after postoperative intestinal adhesion in rats. (a) Plasma IL-6, (b) IL-17F, (c) IL-1*β*, and (d) TNF-*α* in peritoneal fluid levels were measured by ELISA assay at 6 h, 12 h, and 1 week after operation. *∗∗*P<0.01, *∗∗∗*P<0.001 compared with the control group. ###P<0.001 compared with the sham group.

The levels of IL-6 in plasma and IL-*β*1, TNF-*α*, and IL-17F levels in peritoneal fluid were measured by ELISA assay at 6 h, 12 h, and 1 week after operation. The results showed that the levels of IL-6 in plasma and IL-17F in peritoneal fluid in the control group were significantly higher than those in the sham operation group at 6h. Compared with the control group, both* Bletilla striata* and HA inhibited the secretion of IL-6 in plasma and IL-17F in peritoneal fluid (P<0.01) (Figure 4). There was no significant difference of IL-1*β* and TNF-*α* levels in rat's peritoneal fluid between the control group and the sham operation group, which is consistent with the findings of Chegini's research [19].

### 3.5. The Effect of* Bletilla striata* on Postoperative Intestinal Adhesion Is Closely Related to TGF-*β* Signaling Pathway

**Figure 5:**
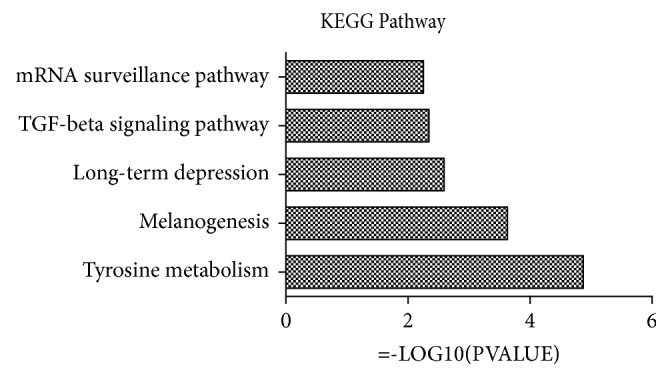
The effect of* Bletilla striata* on postoperative intestinal adhesion is closely related to TGF-*β* signaling pathway. We used TCMID to analyze the biopathway enrichment of the active ingredients of Bletilla striata in the Kyoto Encyclopedia of Genes and Genomes (KEGG) and prove that TGF-*β* signaling pathway was one of the most significant enrichment pathways for BS.

We used TCMID to analyze the biopathway enrichment of the active ingredients of* Bletilla striata* in the Kyoto Encyclopedia of Genes and Genomes (KEGG) and found that mRNA surveillance pathway, TGF-*β* signaling pathway, long-term depression, melanogenesis, and tyrosine metabolism were the enrichment pathways for* Bletilla striata* (Figure 5), so we confirm that TGF-*β* signaling pathway is closely related to the efficacy of* Bletilla striata*.

### 3.6. *Bletilla striata* Inhibited the Levels of TGF-*β*1 in the Cecum after PPAs in Rats

**Figure 6:**
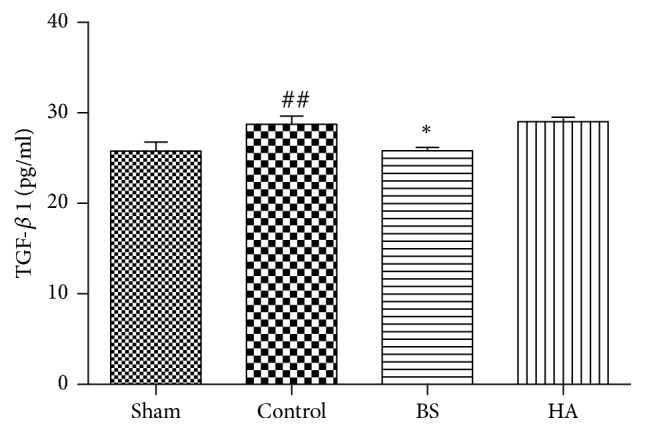
*Bletilla striata* inhibited the levels of TGF-*β*1 in the cecum tissue after postoperative intestinal adhesion in rats. *∗*P<0.05 compared with the control group. ##P<0.05 compared with the sham group (n=8).

The concentration of TGF-*β*1 in cecum tissue was detected by ELISA after PPAs in rats. The results showed that TGF-*β*1 in the control group was significantly higher than that in the sham operation group (P<0.01). The secretion of TGF-*β*1 in* Bletilla striata* group was significantly lower than that in control group (P < 0.01). The secretion of TGF-*β*1 in HA group was not significantly different from that in control group (P > 0.05) (Figure 6). These results show that* Bletilla striata* can prevent adhesion better than HA.

### 3.7. *Bletilla striata* Reduced the Collagen Expression in Cecum Tissue

**Figure 7:**
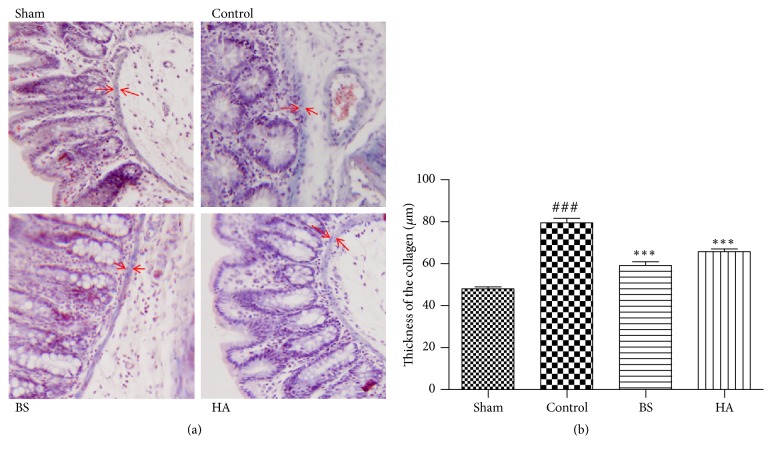
*Bletilla striata* reduced the collagen expression in cecum tissue on the 7th day after operation. (a) The typical image of collagen expression among sham laparotomy, control, BS, and HA group by Masson's trichrome stain. (b) Analysis of collagen thickness by Masson's trichrome stain. *∗∗∗*P<0.01 compared with the control group. ###P<0.001 compared with the sham group.

We examined the thickness of the collagen by Masson's trichrome stain. The blue stained collagen fibers could be detected within mucosal muscularis in sham operation group. In control group, the collagen fibers significantly increased around the mucosal muscularis and submucous layer.* Bletilla striata* and HA could significantly decrease the thickness of collagen fibers in the caecum of rats (Figure 7).

### 3.8. *Bletilla striata* Inhibited the Protein Expression of TGF-*β*1 and *α*-SMA in the Cecum Tissue after PPAs

**Figure 8:**
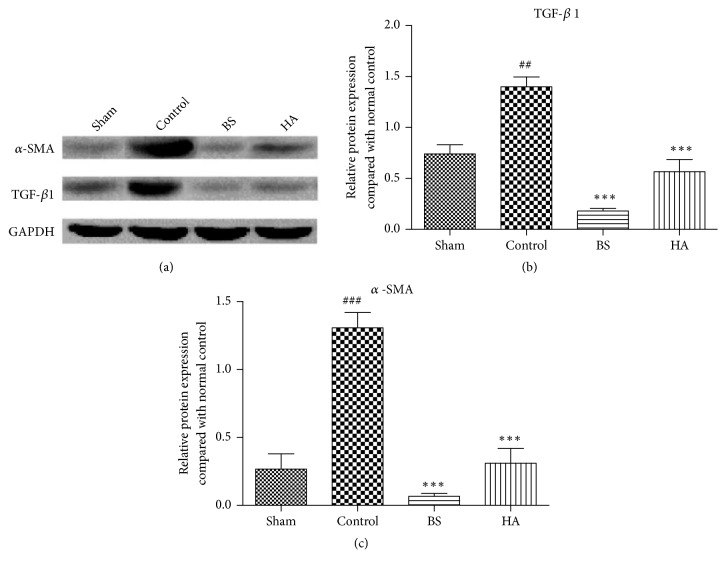
*Bletilla striata* inhibited the protein expression of TGF-*β*1 and *α*-SMA in the cecum tissue after postoperative intestinal adhesion in rats. (a) TGF-*β*1 and *α*-SMA protein bands of Western blot; (b) TGF-*β*1 protein expression; (c) *α*-SMA protein expression. *∗∗∗*P<0.001 compared with the control group. ##P<0.01, ### P<0.001 compared with the sham group (n=3).

We used the TCMID system to analyze TGF-*β* signaling pathway and confirm that it is closely related to the preventing efficacy of* Bletilla striata* on PPAs, and so the key markers of epithelial-mesenchymal transition of *α*-SMA and TGF-*β*1 were determined by Western blotting. The results showed that *α*-SMA and TGF- *β* protein expression significantly increased in the rats of control group, and* Bletilla striata* and HA significantly decreased the protein expression of *α*-SMA and TGF- *β* (P<0.01) (Figure 8).

## 4. Discussion

The aim of this study was to evaluate the importance of* Bletilla striata* in preventing PPAs in rats. The current study found that* Bletilla striata*, a widely used astringent hemostatic medicinal plant, can effectively prevent PPAs by observing the degree of abdominal adhesion on the 7th day after operation. Hyaluronic acid (HA) is an anionic, nonsulfated glycosaminoglycan distributed in connective tissue [20–22]. Previous studies have pointed to the importance of HA in preventing the PPAs [12, 23]. Therefore, in our study, the hyaluronic acid (HA) as a positive drug was used for the treatment of postoperative intestinal adhesion, and it was compared with* Bletilla striata* to observe their effect on the intestinal adhesion after operation.

Previous research has confirmed that inflammation of the entire peritoneum cavity is an important mechanism involved in adhesion formation [24]. Inflammatory reaction is one of the important pathophysiological processes leading to epithelial-mesenchymal transition [25]. Therefore we used the inflammation grading scale to compare inflammatory responses in four groups of rats [14]. The results showed that the inflammatory reaction in* Bletilla striata* group was significantly reduced compared with the control group, indicating that* Bletilla striata* could obviously reduce the inflammatory reaction and prevent intestinal adhesion after operation.

In this experiment, we tested a variety of inflammatory factors to further study the inflammatory response. Concentrations of IL-6, IL-1*β*, and TNF-*α* were changed in plasma and peritoneal fluid at various time points after incision [26]. In our study, concentrations of IL-6 in plasma and IL-17F, IL-1*β*, and TNF-*α* in peritoneal fluid were measured by ELISA assay at 6 h, 12 h, and 1 week after operation. The results showed that there were significant differences between the groups only at 6 hours, and* Bletilla striata *and HA could inhibit the secretion of IL-6 in plasma at 6 hours and have no effect at 12 h and 1 week after operation. This result is consistent with that found by Fredriksson [26]. Surprisingly, the ELISA results of this study showed no significant difference in the concentration of IL-1*β* and TNF-*α* among the four groups at 6 h, 12 h, and 1 week after operation, but this finding was consistent with that of Chegini [19]. Chegini explained that the celiac fluid content of the two cytokines was not related to the formation of peritoneal adhesion. Our study shows that IL-1*β* and TNF-*α* in peritoneal fluid cannot well reflect the severity of PPAs. Wang et al. have confirmed that IL-17F enhances the response of fibroblasts to TGF-*β* and is associated with adhesion formation [8]. The study of Chung found that IL-17F, a proinflammatory cytokine derived from T cells, showed a key role in adhesion formation [27]. Our study found that* Bletilla striata* could inhibit the secretion of IL-17F in peritoneal fluid at 6 hours and has no effect at 12 h and 1 week after operation.

We still conducted an immunohistochemistry experiment to test TNF-*α* level in cecum tissue because TNF-*α* has already been demonstrated as a good biological marker for PPAs, although TNF-*α* in peritoneal fluid is not a sensitive indicator [28]. Immunohistochemical results showed that TNF-*α* expressing cells were significantly greater in control group than in sham operation group. In* Bletilla striata* group, the expression of TNF-*α* was low in caecum. The inhibitory effect of Bletilla striata on PPAs was further proved.

We used the TCMID system to analyze TGF-*β* signaling pathway and confirm that it is closely related to the preventing efficacy of* Bletilla striata* on PPAs. TGF-*β*, a substance that is expressed by all cell types, is thought to be a key factor in all stages of the wound healing process and is the major profibrotic cytokine. It can induce and be responsible for the activation of epithelial-mesenchymal transitions (EMTs). EMT is the process by which epithelial cells transform into mesenchymal cells and will eventually cause organ fibrosis [25, 29]. TGF-*β* will induce cell differentiation into myofibroblasts, which is highly associated with fibrotic diseases and will upregulate the expression of *α*-SMA and collagens [30, 31]. The action of TGF-*β* in tissue fibrosis appears to occur predominately through overexpression of TGF-*β*1 compared with TGF-*β*2 and TGF-*β*3 isoforms [32]. Our study showed that* Bletilla striata* inhibited the levels of TGF-*β*1 in the cecum tissue after postoperative intestinal adhesion in rats by ELISA and significantly decreased the thickness of collagen fibers in the caecum of rats by Masson's trichrome stain. Moreover* Bletilla striata* significantly decreased the protein expression of *α*-SMA and TGF-*β* by Western blotting. These results suggest that* Bletilla striata* could reduce the expression of the important substance which increased in PPAs.

The results of Figure 6 showed that HA could not significantly inhibit the secretion of TGF-*β*1 compared with the control group, meaning that HA cannot completely prevent all of the pathological processes of adhesion. Wei et al. [33] have proved that HA could not inhibit the expression of TGF-*β* in peritoneal adhesion model of rats at the eighth day. Beyond that, a meta-analysis of four trials involving the use of hyaluronic acid [34–37] found no difference in overall mean adhesions scores [21]. A powder form containing hyaluronic acid was found to produce more adverse events [38]. These results further illustrate the importance of developing new drugs against surgical adhesion.* Bletilla striata* is an important astringent hemostatic medicine used for almost 2000 years in China and it has little side effect. At the same time, this study proved that* Bletilla striata* can effectively prevent the formation of adhesion after operation in rats, which is related to the inhibition of inflammatory factor IL-17F, IL-6, and TNF-*α* in different parts of rats, and can also prevent the expression of TGF-*β*, *α*-SMA, and collagen fibers. All this indicates that* Bletilla striata* has potential antiadhesion effect and is worthy of further study to be developed into a clinical drug for antiadhesion after operation.

## Data Availability

The data used to support the findings of this study are available from the corresponding author upon request.
